# Epidemiology of Bluetongue Virus Serotype 8, Germany

**DOI:** 10.3201/eid1503.081210

**Published:** 2009-03

**Authors:** Franz J. Conraths, Jörn M. Gethmann, Christoph Staubach, Thomas C. Mettenleiter, Martin Beer, Bernd Hoffmann

**Affiliations:** Friedrich-Loeffler-Institut, Wusterhausen, Germany (F.J. Conraths, J.M. Gethmann, C. Staubach); Friedrich-Loeffler-Institut, Greifswald-Insel Riems, Germany (T.C. Mettenleiter, M. Beer, B. Hoffmann)

**Keywords:** Bluetongue disease, serotype 8, ruminants, Culicoides, epidemiology, RT-PCR, mortality, dispatch

## Abstract

In Germany, bluetongue disease had not been reported before 2006. During August 2006–August 2008, >24,000 bluetongue virus serotype 8 infections were reported, most (20,635) in 2007. In 2006 and 2007, respectively, case-fatality rates were 6.4% and 13.1% for cattle and 37.5% and 41.5% for sheep. Vaccination in 2008 decreased cases.

Bluetongue disease (BT) is an infectious, but noncontagious, viral infection of ruminants that is transmitted by *Culicoides* spp. biting midges. It can cause massive losses in farmed ruminants, particularly sheep. BT had never been reported in Europe north of the Alps before August 2006, when outbreaks almost simultaneously occurred in Belgium, France, Germany, and the Netherlands (*1*–*4*). We examined the epidemiology of this outbreak in Germany, where reporting of BT cases through the national animal disease notification system (*5*) is mandatory. In this article, an outbreak is defined as the occurrence of BT in cattle, sheep, or goats on a farm involving at least 1 infected animal; a case is defined as a single infected animal. Wild ruminants infected with BT were always considered cases.

## The Study

In Germany, cattle were tested for BTV before trade or if disease was suspected from clinical signs. Diagnoses were made by serologic testing with commercial test kits (Pourquier ELISA Bluetongue Serum, Institut Pourquier, Montpellier, France; Bluetongue Virus Antibody Test Kit, VMRD, Pullman, WA, USA; ID Screen Blue Tongue Competition ELISA Kit, ID Vet, Montpellier, France; INGEZIM BTV, INGENASA, Madrid, Spain) or real-time reverse transcription–PCR (RT-PCR) (*6*).

Because no real-time RT-PCR for the sensitive detection of BTV serotype 8 (BTV-8) was available in Europe in 2006, we developed and validated such an assay. For the detection of BTV-8 genome, 0.8-µM primers (BTV8-NS1-1F [5′-AAT GGG ATG TGT GTC AAA CAA AAT-3′]; BTV8-NS1-1R [5′-CAA CTA ATT TAT ACG CTT TCG CC-3′]) and a 0.2-µM probe (BTV8-NS1-1FAM [5′-FAM-CTC CTC CGC ATC GGT CGC CGC-TAMRA-3′]) were used in a QuantiTect Probe RT-PCR kit (QIAGEN, Hilden, Germany). The method was then transferred to and implemented at the regional laboratories of the German federal states responsible for BT screening; the results were confirmed by proficiency testing. Since the summer of 2007, real-time RT-PCR assays for all known 24 serotypes of BTV (*3*) have also been used for BTV genome detection. Samples with inconclusive results in the differential pan-BTV and BTV-8 tests were referred to the national reference laboratory, which confirmed the exclusive presence of BTV-8 during the study period (August 2006–August 2008).

**Figure 1:**
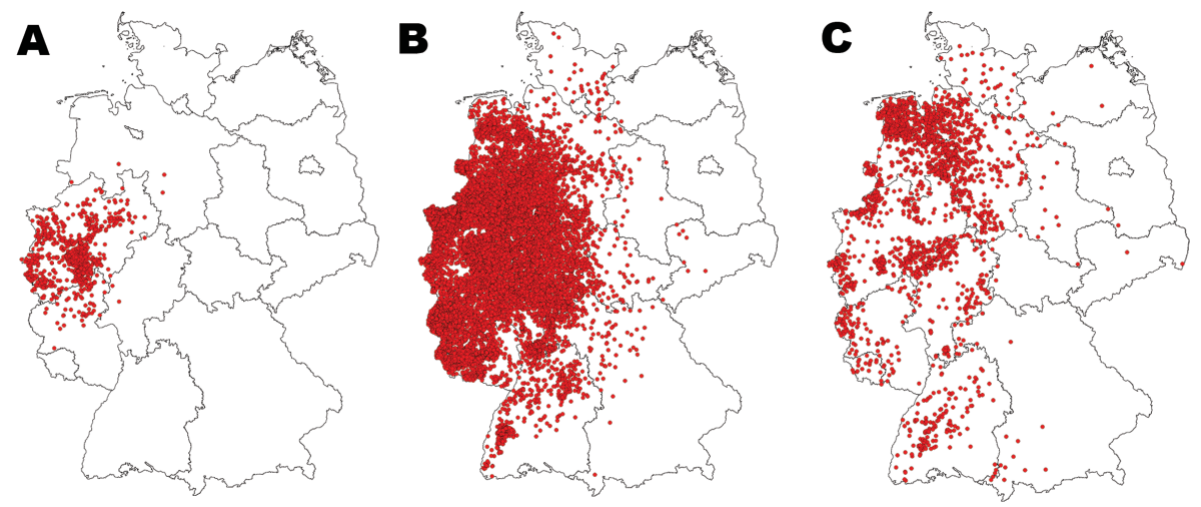
Maps showing outbreaks of bluetongue disease among all affected species in Germany in A) 2006, B) 2007, and C) 2008 (through August 31). Red dots indicate confirmed cases/outbreaks.

**Figure 2:**
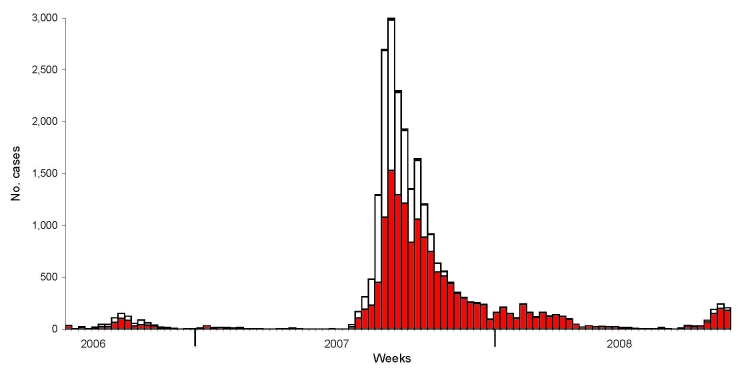
Number of new cases/outbreaks of bluetongue disease per calendar week in cattle (red), sheep (white), and goats (black), Germany.

In 2006, the disease was diagnosed on 571 cattle farms and in 309 sheep flocks, 3 other bovines, 6 red deer, 3 mouflons, and 1 roe deer in the federal states of North Rhine-Westphalia, Rhineland-Palatinate, Hesse, Lower Saxony, and Saarland (Figure 1, panel A). The core region of the epidemic in Germany was in North Rhine-Westphalia, adjacent to the affected areas in Belgium, the Netherlands, and Luxembourg. The disease was first detected in late August in calendar week 34 (37 cases), peaked mid October in calendar week 42 (154 cases), and decreased slowly until the end of the year (Figure 2). Apparently, the infection overwintered in the region (*6*) and flared up again in 2007, spreading over most of Germany during the summer and autumn of 2007 and resulting in 20,624 new outbreaks (Figure 1, panel B). During 2007, BT was detected on 12,638 cattle farms and in 23 other individual bovines, 7,790 sheep flocks, 115 goat herds, 34 fallow deer, 11 mouflons, 10 red deer, and 3 roe deer. The first case of 2007 was detected in early June, calendar week 23. The number of new cases started to rise constantly from the end of July (week 30), peaked at 3,001 new cases in mid September (week 37), and subsequently decreased slowly until the end of year. The number of affected animals was higher for sheep in the first months of the BT season in 2007; the number of affected cattle dominated the late phase (Figure 2).

In 2006, at least 48,364 cattle, 9,781 sheep, and 56 goats were exposed to BTV on affected premises (Table). Of these animals, 1,131 cattle (2.34%) and 590 sheep (6.03%) were found to be infected; 72 cattle and 221 sheep died. The case-fatality rate was much higher for sheep (37.5%) than for cattle (6.4%). These calculations are based on the assumption that all BT cases were reported. Because the infections caused only mild disease or remained even clinically inapparent in some animals, especially cattle, underreporting is likely and the case-fatality rate in cattle may be slightly overestimated.

**Table:** Animals affected by bluetongue virus serotype 8, Germany, 2006 and 2007

Animals	Total no. animals	No. diseased animals	No. deaths	Morbidity rate, %	Mortality rate, %	Case-fatality rate, %
Cattle						
2006	48,364	1,131	72	2.34	0.15	6.37
2007	1,317,111	26,772	3,512	2.03	0.27	13.12
Sheep						
2006	9,781	590	221	6.03	2.26	37.46
2007	503,282	32,116	13,324	6.38	2.65	41.49
Goats						
2006	56	0	0	0	0	0
2007	3,346	209	54	6.25	1.61	25.84

In 2007, due to the spread of the disease, the exposed population rose to at least 1,317,111 cattle, 503,282 sheep, and 3,346 goats on affected farms. The numbers of infected animals on these farms amounted to 26,772 cattle, 32,116 sheep, and 209 goats in 20,624 new outbreaks. While mortality rates remained relatively low, as in 2006, the case-fatality proportion rose to 13.1% in cattle and 41.5% in sheep.

In 2008, BT incidence decreased considerably under the influence of a mass vaccination campaign that started in May and June 2008 (Figure 2), before the 2008 vector season. In 2008, a total of 1,070 new cases (PCR-positive, sampled after May 1, 2008; i.e., infection was acquired during the current transmission season) were reported. They were found mainly in 2 regions in northwestern Lower Saxony and western Baden-Württemberg (Figure 1, panel C), where the vaccination campaign started relatively late because of an initially limited supply of BTV-8-vaccine.

## Conclusions

The number of BTV-8 infections in Germany that peaked during the summer and autumn of 2007 showed that even a limited BTV-8 outbreak can dramatically spread within a few months after detection of the first cases. After the initial incursion and limited spread in 2006, BTV-8 overwintered, resulting in efficient spread of BTV-8 during 2007 and severe consequences for cattle and sheep farmers. The case-fatality rate was ≈3× higher for sheep than for cattle (37.5% vs. 6.4% in 2006 and 41.5% vs. 13.1% in 2007). These findings illustrate that BTV-8 was more pathogenic for sheep than for cattle. It must be stressed, however, that the virus caused clinical disease and death in cattle, although other serotypes cause clinical disease and deaths primarily in sheep.

As a result of the vaccination campaign, the number of new cases reported in 2008 (through August 2008) was substantially lower than in 2007, demonstrating that convalescent animals are protected from reinfection and that vaccination was successful. In addition, restriction of animal movement from protection and surveillance zones appears reasonable, although the ability of restricted movement to decrease virus spread is limited, particularly during the vector season.
